# H_2_S Donor NaHS Changes the Production of Endogenous H_2_S and NO in D-Galactose-Induced Accelerated Ageing

**DOI:** 10.1155/2017/5707830

**Published:** 2017-04-23

**Authors:** Wei Wu, Cui-Lan Hou, Xue-Pan Mu, Chen Sun, Yi-Chun Zhu, Ming-Jie Wang, Qian-Zhou Lv

**Affiliations:** ^1^Department of Pharmacy, Zhongshan Hospital, Fudan University, Shanghai 200032, China; ^2^Department of Physiology and Pathophysiology, School of Basic Medical Sciences, Shanghai Key Laboratory of Clinical Geriatric Medicine, Fudan University, Shanghai 200032, China

## Abstract

*Aims*. The study was designed to explore whether hydrogen sulphide (H_2_S) and nitric oxide (NO) generation changed in D-galactose- (D-gal-) induced ageing, the possible effects of exogenous H_2_S supplementation, and related mechanisms. *Results*. In D-gal-induced senescent mice, both H_2_S and NO levels in the heart, liver, and kidney tissues were decreased significantly. A similar trend was observed in D-gal-challenged human umbilical vein endothelial cells (HUVECs). Sustained H_2_S donor (NaHS) treatment for 2 months elevated H_2_S and NO levels in these mice, and during this period, the D-gal-induced senescent phenotype was reversed. The protective effect of NaHS is associated with a decrease in reactive oxygen species levels and an increase in antioxidants, such as glutathione, and superoxide dismutase and glutathione peroxidase activities. Increased expression of the H_2_S-producing enzymes cystathionine *γ*-lyase (CSE) and cystathionine-*β*-synthase (CBS) in the heart, liver, and kidney tissues was observed in the NaHS-treated groups. NaHS supplementation also significantly postponed D-gal-induced HUVEC senescence. *Conclusions*. Endogenous hydrogen sulphide production in both ageing mice and endothelial cells is insufficient. Exogenous H_2_S can partially rescue ageing-related dysfunction by inducing endogenous H_2_S and NO production and reducing oxidative stress. Restoring endogenous H_2_S production may contribute to healthy ageing, and H_2_S may have antiageing effects.

## 1. Introduction

With advancements in medical care, the population of people over 65 years old is growing rapidly. However, age-related health deterioration has not received enough attention, which may pose new problems for the healthcare system [1]. Ageing is characterized by the progressive loss of physiological functions and is regarded as the main risk factor for the development of age-related diseases such as hypertension, coronary atherosclerosis, type 2 diabetes, and neurodegenerative disorders [1–4]. To understand the causes and mechanisms of ageing and age-related diseases is therefore of great importance.

Low-dose administration of D-galactose (D-gal) in vitro (1 to 100 g/L, 48 hours) and in vivo (125 mg/kg/day, 8 weeks) is widely accepted as an experimental model for the study of accelerated ageing [5–8]. D-gal exposure results in oxidative stress inducing ageing, which resembles the natural ageing process in mice [9].

Cellular senescence can be postponed by scavenging intracellular reactive oxygen species (ROS), and cell survival can thus be improved [10, 11]. Given that H_2_S regulates several key proteins involved in cellular oxidative stress, it could have a protective effect against ageing. For example, H_2_S induces the S-sulphydration of Keap1, which leads to Nrf2 activation and nuclear translocation and results in the synthesis of antioxidative proteins [12]. H_2_S also inhibits mitochondrial ROS production and prevents activation of the adaptor protein p66Shc [13]. Higher oxidative stress is often accompanied with less nitric oxide (NO) production, which may weaken the cardio-protecting effect of NO [14].

Recently, studies have concentrated on the possibility of lifespan extension by administrating exogenous H_2_S or manipulating its endogenous production. The regulatory role of exogenous H_2_S in *C. elegans* ageing was first reported by Miller and Roth [15] and later confirmed by Wei and Kenyon [16]. In mouse embryonic fibroblasts, CSE deficiency led to an early development of cellular senescence [12], indicating the protective effect of endogenous H_2_S in senescence. Our previous study reported decreased heart H_2_S levels in long-term fructose-fed ageing mice, which may contribute to the pathogenesis of diabetic cardiomyopathy [17]. We also found that H_2_S protected the ageing kidney by alleviating oxidative stress, inducing endogenous H_2_S production and Nrf2 nuclear translocation [18]. However, the relationship between the endogenous production of two gasotransmitters, H_2_S and NO, during the ageing process is not clear. Moreover, whether H_2_S supplementation manipulates NO production during ageing and whether it has a protective effect against ageing-related tissue damage is far from well understood. The present study aims to study the effect and possible mechanism of the action of H_2_S on cellular senescence during accelerated ageing both in vitro and in vivo.

## 2. Materials and Methods

### 2.1. Drugs and Accelerated Ageing Protocol

D-Galactose was purchased from Sigma-Aldrich (Germany). Eight-week-old male C57BL/6 mice were obtained from the Department of Laboratory Animal Science of Fudan University and raised under controlled conditions (22 ± 2°C, 45–55% relative humidity, and 12 h dark-light cycle). A total of 60 mice were randomly assigned to the control group with normal saline and four D-gal model groups with hypodermic injections of 50 mg/kg D-gal daily for 2 months. The mice in each D-gal model group received different intraperitoneal injections of NaHS (Sigma-Aldrich, Germany) or normal saline once a day at the same time: D-gal with normal saline, D-gal with low-dose NaHS (10 *μ*mol/kg/day), D-gal with medium-dose NaHS (50 *μ*mol/kg/day), and D-gal with high-dose NaHS (100 *μ*mol/kg/day). All animal studies were approved by the Ethics Committee of Experimental Research, Fudan University Shanghai Medical College. For in vitro studies, subacute senescence was induced in human umbilical vein endothelial cells (HUVECs) by incubating cells with 50 mmol/L D-gal for 48 h [8]. Different doses of NaHS were added 24 h after the onset of the D-gal challenge.

### 2.2. Measurement of H_2_S Levels

H_2_S levels in the plasma, heart, liver, and kidney tissues were determined as previously described [19]. Briefly, 30 *μ*L of homogenized heart, liver, or kidney tissues were incubated with 80 *μ*L of monobromobimane (MBB) for 40 min on a shaker at room temperature. The reaction was terminated by adding 20% formic acid, and H_2_S levels were measured by a gas chromatograph-mass spectrometer (Leica, Germany).

### 2.3. Measurement of NO Levels

An NO assay kit (Beyotime Biotechnology, China) was used to measure nitrite/nitrate (NOx) via the Griess reaction. Nitrate was converted into nitrite by using nitrate reductase, and then, nitrite was measured.

### 2.4. Detection of ROS Levels

ROS levels in the heart tissue and HUVECs were measured by using dihydroethidium (DHE) staining (Sigma-Aldrich, Germany). Mouse heart tissue sections (7 *μ*m) were obtained by using a frozen tissue slicer. Heart tissue sections or HUVECs were incubated with 10 nmol/L DHE for 30 min at 37°C and observed under a laser confocal microscope (Zeiss LSM710) at the excitation/emission wavelengths of 488/610 nm. Fluorescence values were normalized to the control groups.

### 2.5. Morphological and Histological Analyses

The kidney tissues were excised, fixed in 10% formalin, and embedded in paraffin. Kidney sections (4 *μ*m) were stained with haematoxylin and eosin (HE). eNOS immunohistochemical staining was performed according to the manufacturer's instructions. Renal pathological changes were observed under an optical microscope (Olympus, Japan).

### 2.6. Senescence-Associated *β*-Galactosidase (SA-*β*-gal) Staining

The percentage of SA-*β*-gal positive cells was determined by using a senescence *β*-galactosidase staining kit (Beyotime Biotechnology, China). Briefly, HUVECs cultured in 12-well plates were fixed and washed with phosphate-buffered saline (PBS) thrice. Cell-staining working solution (0.5 mL) was added into each well and incubated at 37°C for 12 h. After washing with PBS, senescent cells were identified as blue-stained cells under an inverted microscope (Leica, Germany). For each well, a minimum of 500 cells was counted to determine the percentage of SA-*β*-gal positive cells.

### 2.7. Cell Proliferation Assay

Cell proliferation was measured by the Cell Counting Kit-8 (CCK-8) (Beyotime Biotechnology, China) assay. Briefly, HUVECs were cultured in 96-well plates, exposed to D-gal for 46 h and various dosages of NaHS for 22 h. Subsequently, the cell culture medium was replaced with CCK-8 buffer (10 *μ*L CCK-8 in 100 *μ*L PBS, Beyotime Biotechnology, China) and incubated in a CO_2_ incubator for 2 h. Afterward, cells were washed thrice with PBS, and the optical density (OD) in each well was determined by using a microplate reader at 450 nm.

### 2.8. Western Blot Analysis

Proteins from the heart, liver, kidney tissues, and HUVECs were collected and quantified by using the BCA reagent (Shen Neng Bo Cai Corp., China). Protein samples were loaded on a sodium dodecyl sulphate polyacrylamide gel (10%), separated through electrophoresis, transferred onto a polyvinylidene fluoride membrane (Millipore, Bedford, MA), and incubated with primary antibodies (1 : 1000 dilution) against CSE, CBS, 3-MST (Santa Cruz Biotechnology, CA), p-eNOS, eNOS (BD Company, USA), p16, p21, or p53 (Proteintech, China) at 4°C overnight. The blots were washed thrice with Tris-buffered saline containing Tween 20 (TBST) and incubated with horseradish peroxidase-conjugated secondary antibodies for 1 h at room temperature. The blots were then washed thrice and visualized by using chemiluminescent substrate (ECL). The densities of the immunostained bands were analysed by using a scanning densitometer (model GS-800, Bio-Rad) and Bio-Rad's image analysis software.

### 2.9. Real-Time PCR Analysis

Total RNA from HUVECs was prepared using Trizol reagent (Sheng Neng Bo Cai Corp., China) according to the manufacturer's instructions. cDNA was generated from 0.5–2 *μ*g total RNA using a reverse transcription kit (Toyobo, Japan). Real-time PCR was tested using the SteponePlus Real-Time PCR System (Applied Biosystems, USA) in a total volume of 20 *μ*L reaction mixture containing 2 *μ*L cDNA, 10 *μ*L 2× SYBR Green PCR Master Mix (Toyobo, Japan), 0.8 *μ*L of each primer (10 *μ*M), and 6.4 *μ*L ddH_2_O. Three-step real-time PCR of denaturing, annealing, and extension reactions was performed for 40 cycles of 10 s at 95°C, 30 s at 60°C, and 20 s at 72°C. For the eNOS gene, the forward primer was 5′-AGGTCTGTGGGTCTGGTTTG-3′ and the reverse primer was 5′-GCTCATTCTCCAGGTGCTTC-3′. For the GAPDH gene, the forward primer was 5′-CCACCCATGGCAAATTCC-3′ and the reverse primer was 5′-GATGGGATTTCCATTGATGACA-3′.

### 2.10. Statistical Analysis

Results are expressed as the means ± SEM. Statistical analysis was performed by using SPSS software, version 21.0 (SPSS Inc., Chicago, IL, USA). Comparisons among groups were performed by one-way ANOVA. Paired data were evaluated by two-tailed Student's *t*-test. Differences were considered statistically significant when *P* < 0.05.

## 3. Results

### 3.1. NaHS Mitigated the Decreased Endogenous Production of H_2_S and NO in D-gal-Treated Mice and HUVECs

**Figure 1:**
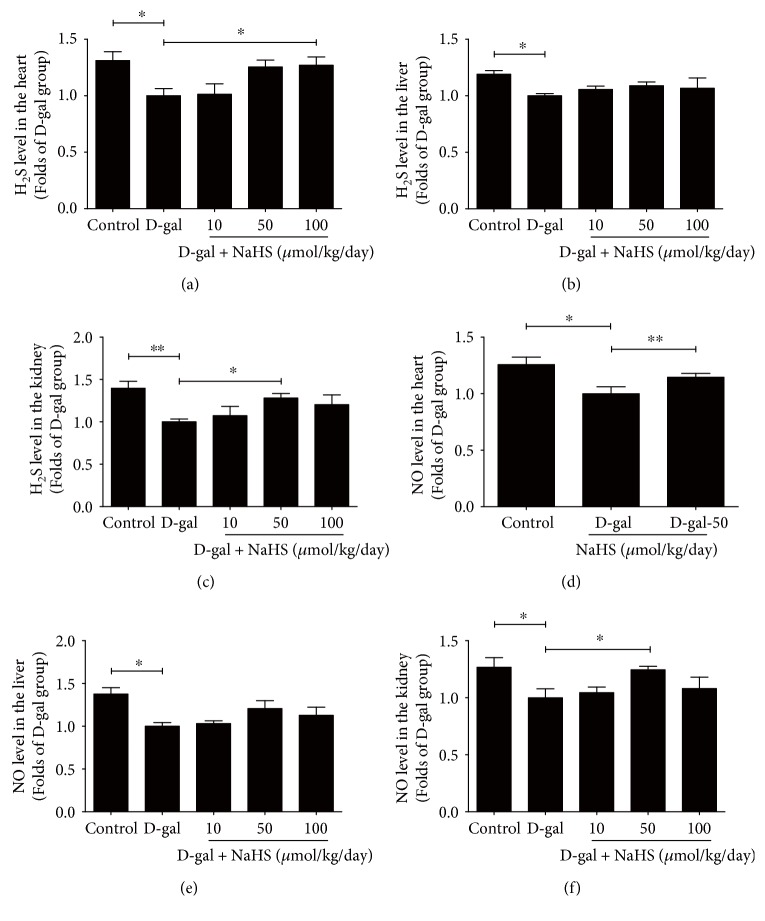
H_2_S and NO levels in ageing mice. (a) H_2_S levels in the heart tissues. (b) H_2_S levels in the liver tissues. (c) H_2_S levels in the kidney tissues. (d) NO levels in the heart tissues. (e) NO levels in the liver tissues. (f) NO levels in the kidney tissues. *N* = 6 for H_2_S measurements and *N* = 8 for NO measurements. Values are the means ± SE. ^∗^*P* < 0.05 and ^∗∗^*P* < 0.01.

Compared with the control group, we found that in the heart, liver, and kidney, H_2_S levels decreased significantly in the D-gal model group. NaHS supplementation for 8 weeks mitigated this decrease. However, the optimal NaHS concentration was not identical in different tissue types. In the heart, 100 *μ*mol/kg/day of NaHS was the most effective dose, while in the kidney, 50 *μ*mol/kg/day of NaHS was optimum (Figures 1(a), 1(b), and 1(c)).

Compared with the control group, the NO levels were also decreased in D-gal-challenged heart, liver, and kidney tissues. Sustained treatment with NaHS at 50 *μ*mol/kg/day mitigated the reduction in the heart and kidney. In the liver, 50 *μ*mol/kg/day of NaHS increased the NO levels, but the differences were not statistically significant compared to the control group (Figures 1(d), 1(e), and 1(f)).

**Figure 2:**
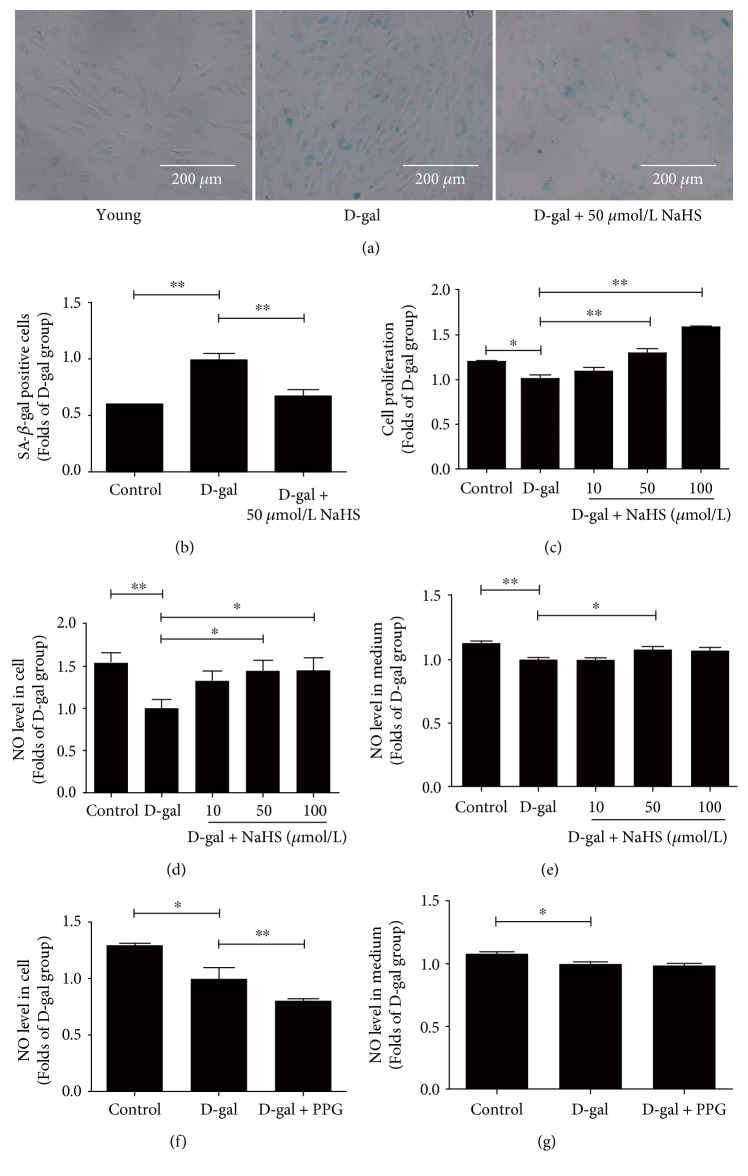
Verification of the in vitro accelerated ageing model and the influence of NaHS treatment on NO levels in ageing HUVECs. (a) Representative senescence-associated *β*-galactosidase (SA-*β*-gal) staining in HUVECs. (b) Statistical analysis of SA-*β*-gal positive cells compared with the D-gal group (*N* = 6). (c) Statistical analysis of cell proliferation determined by the CCK-8 assay (*N* = 10). (d) and (e) NO levels in the lysate and culture medium (*N* = 7). (f) and (g) NO levels in the lysate and culture medium with or without the CSE inhibitor PPG (*N* = 6). Values are the means ± SE. ^∗^*P* < 0.05 and ^∗∗^*P* < 0.01.

D-gal exposure for 48 h augmented the percentage of SA-*β*-gal positive cells, which confirmed the successful establishment of an in vitro accelerated ageing model. Furthermore, 50 *μ*mol/L of NaHS abated senescence (Figures 2(a) and 2(b)) and improved cell proliferation (Figure 2(c)). NO levels inside the cell and in the culture medium were decreased in the D-gal group, but exogenous NaHS supplementation could reverse this trend (Figures 2(d) and 2(e)). HUVECs treated with the CSE inhibitor DL-propargylglycine (PPG) produced even less NO inside the cell, while the amount of NO in the culture medium remained unchanged (Figures 2(f) and 2(g)).

### 3.2. Expression of Three H_2_S-Producing Enzymes in Accelerated Ageing

**Figure 3:**
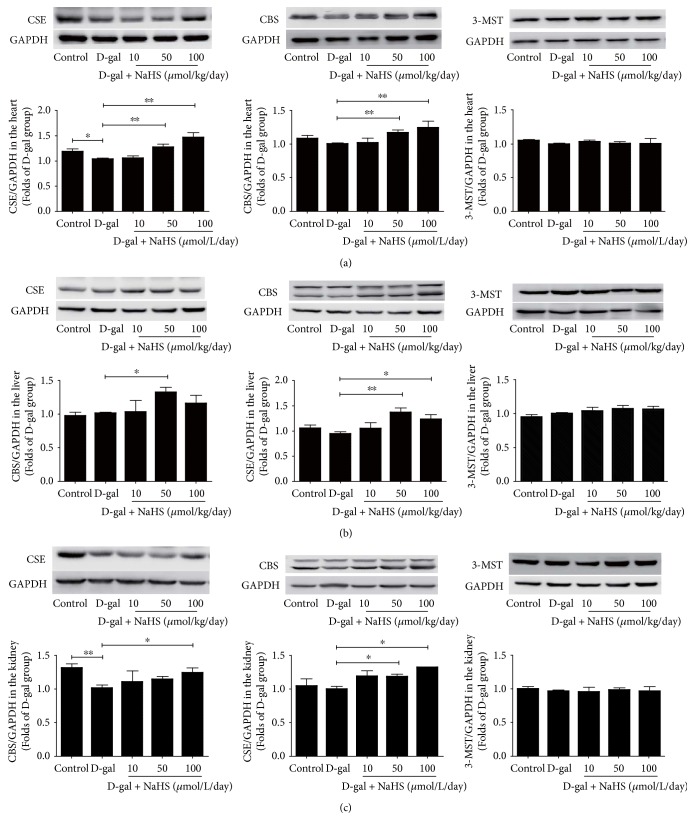
Expression of H_2_S-producing enzymes in different tissues from ageing mice. (a) CSE, CBS, and 3-MST expressions in the heart tissues. (b) CSE, CBS, and 3-MST expressions in the liver tissues. (c) CSE, CBS, and 3-MST expressions in the kidney tissues. *N* = 6. Values are the means ± SE. ^∗^*P* < 0.05 and ^∗∗^*P* < 0.01.

NaHS treatment alleviated the reduction of H_2_S production in D-gal-challenged mice by increasing the expression of H_2_S-producing enzymes. In the heart tissues, compared with the control group, mice treated with D-gal had lower CSE, while 3-MST expression was unchanged. After two months of NaHS treatment (50 and 100 *μ*mol/kg/day), the expressions of both CSE and CBS, but not the 3-MST, increased significantly (Figure 3(a)). In the liver tissues, however, D-gal failed to influence the expression of the three H_2_S-producing enzymes. However, sustained 50 *μ*mol/kg/day of NaHS further increased CSE and CBS expressions. NaHS at 100 *μ*mol/kg/day also mildly increased CBS expression (Figure 3(b)). In the kidney tissues, only CSE expression was decreased upon D-gal exposure. Sustained NaHS therapy at 100 *μ*mol/kg/day increased CSE and CBS expressions (Figure 3(c)).

**Figure 4:**
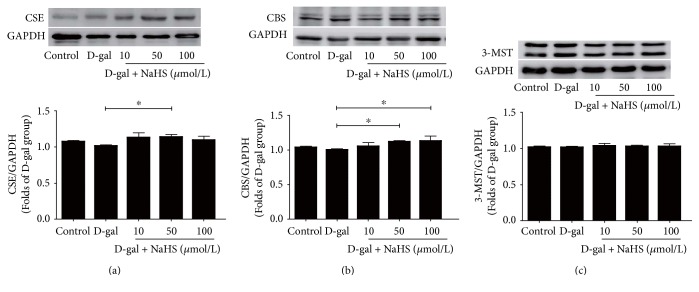
Effects of NaHS treatment on the expression of H_2_S-producing enzymes in ageing HUVECs. (a) CSE expression in HUVECs. (b) CBS expression in HUVECs. (c) 3-MST expression in HUVECs. *N* = 6. Values are the means ± SE. ^∗^*P* < 0.05.

In D-gal-challenged HUVECs, 50 *μ*mol/L of NaHS improved CSE and CBS expressions but failed to alter 3-MST expression (Figure 4).

### 3.3. Histological Changes and eNOS Expression in the Accelerated Ageing Kidney

**Figure 5:**
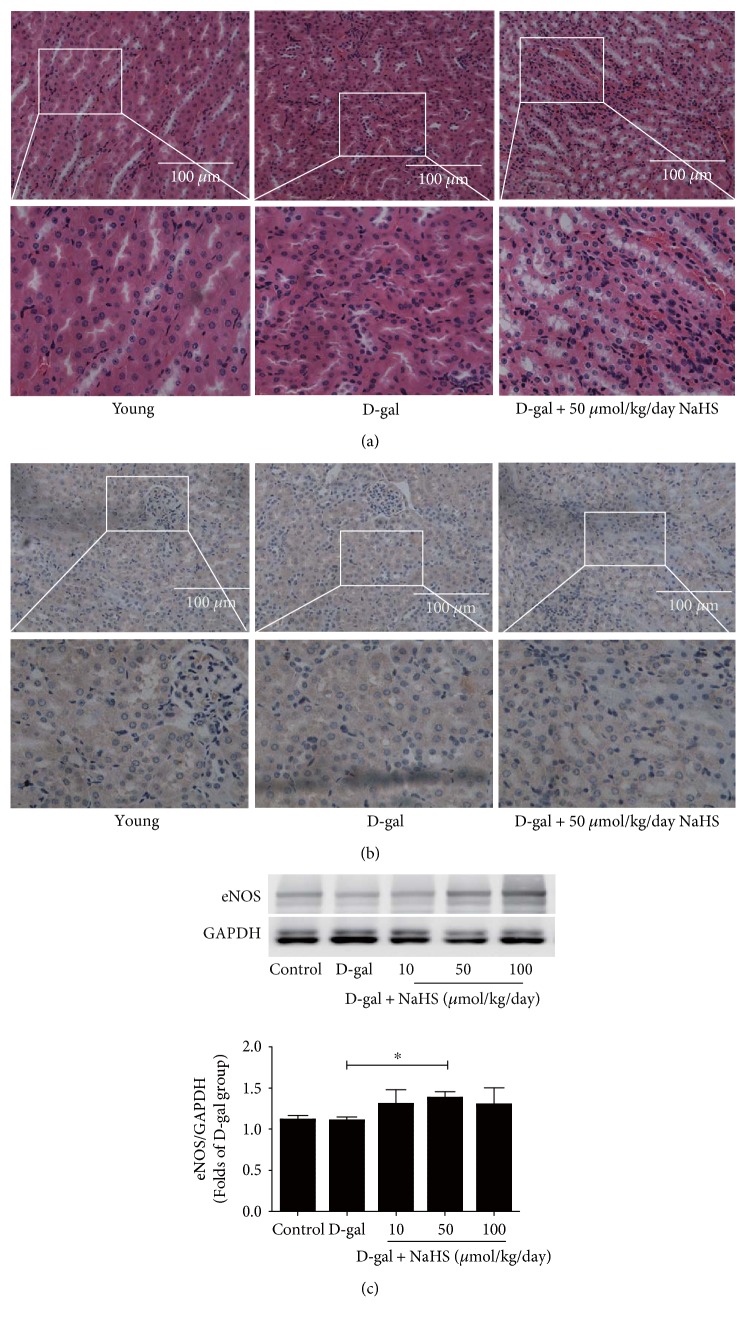
Histological changes and eNOS expression in ageing kidney. (a) Representative HE staining of kidney tissues. (b) Representative immunohistochemical staining of eNOS in kidney tissues. (c) Statistical analysis of eNOS protein expression in kidney tissues. *N* = 5. Values are the means ± SE. ^∗^*P* < 0.05 and ^∗∗^*P* < 0.01.

HE staining of kidney sections revealed tubular regeneration, inflammatory infiltration, and interstitial fibroblast proliferation in accelerated ageing mice. Sustained NaHS therapy could mitigate this damage (Figure 5(a)). Immunohistochemical staining indicated the distribution of eNOS in both the renal tubules and glomeruli (Figure 5(b)). Although no difference in eNOS protein levels was found between the control and D-gal groups, NaHS treatment for two months at 50 *μ*mol/kg/day could induce its expression in the kidney tissues (Figure 5(c)).

### 3.4. NaHS Alleviates Oxidative Stress during Accelerated Ageing

**Figure 6:**
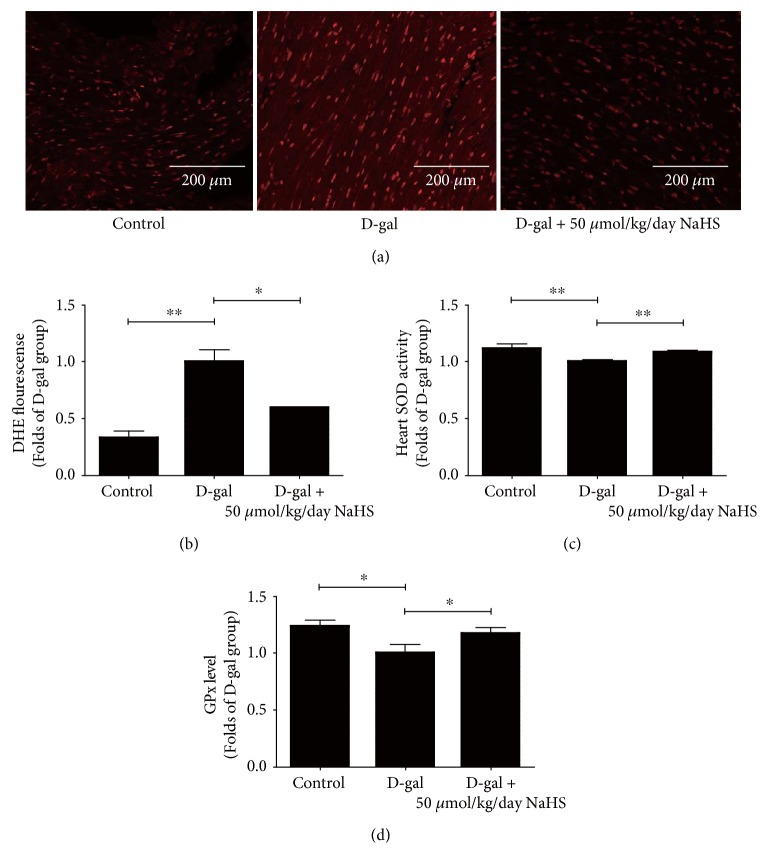
H_2_S donor NaHS protected ageing mice from oxidative stress damage. (a) Representative DHE staining in the heart tissues. (b) Statistical analysis of DHE fluorescence. (c) Total SOD activity in the heart lysate. (d) Glutathione peroxidase (GPx) levels in the heart lysate. *N* = 6. Values are the means ± SE. ^∗^*P* < 0.05 and ^∗∗^*P* < 0.01.

ROS levels in the ageing heart and HUVECs were examined after NaHS therapy. In the heart tissues, compared with the control group, DHE fluorescence intensity was elevated significantly in the D-gal-treated group. Sustained NaHS treatment at 50 *μ*mol/kg/day could mitigate these changes (Figures 6(a) and 6(b)). Accordingly, SOD activity and glutathione peroxidase (GPx) levels were decreased in the D-gal model, and NaHS treatment at 50 *μ*mol/kg/day could partially rescue this decline (Figures 6(c) and 6(d)).

**Figure 7:**
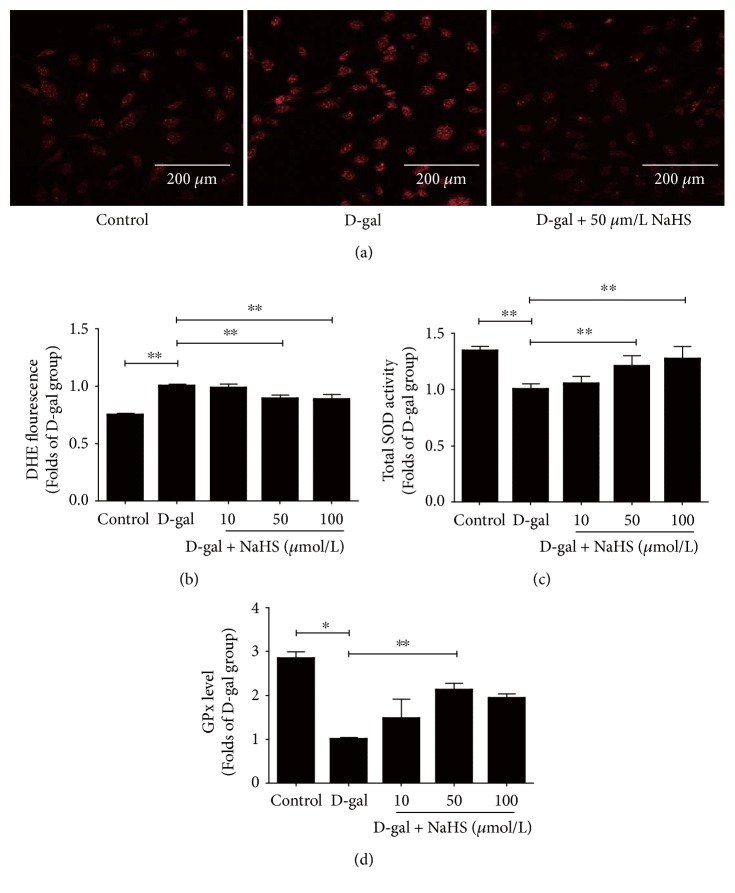
H_2_S donor NaHS protected ageing HUVECs from oxidative stress. (a) Representative DHE staining in HUVECs. (b) Statistical analysis of DHE fluorescence in HUVECs. (c) Total SOD activity. (d) GPx levels in HUVECs. *N* = 6. Values are the means ± SE. ^∗^*P* < 0.05 and ^∗∗^*P* < 0.01.

In HUVECs, DHE fluorescence intensity was increased in the D-gal group (Figures 7(a) and 7(b)). SOD activity and GPx levels were decreased in these cells. NaHS treatment at 50 *μ*mol/kg/day mitigated these changes (Figures 7(c) and 7(d)). Our results indicated that NaHS could protect animals undergoing accelerated ageing and endothelial cells from oxidative stress.

### 3.5. Effects of NaHS Treatment on eNOS Expression and AKT Phosphorylation in D-gal-Treated Senescent HUVECs

**Figure 8:**
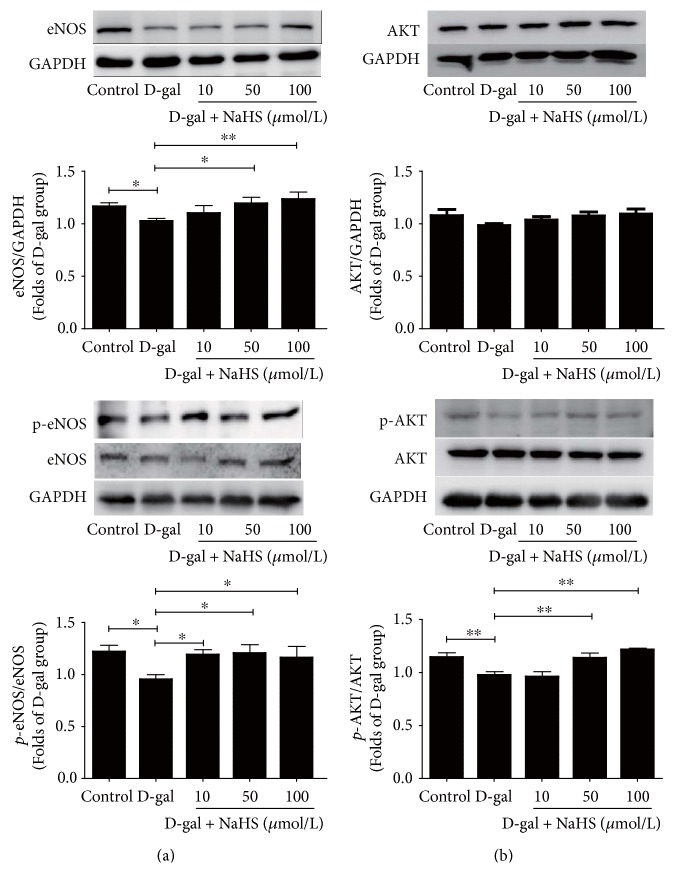
Influence of NaHS treatment on the expression and phosphorylation of AKT and eNOS in ageing HUVECs. (a) NaHS induced both phosphorylation and total eNOS expression in HUVECs. (b) NaHS increased AKT phosphorylation. *N* = 6. Values are the means ± SE. ^∗^*P* < 0.05 and ^∗∗^*P* < 0.01.

Since eNOS is the major NO-producing enzyme in endothelial cells, we checked eNOS expression as well as its phosphorylation in D-gal-challenged HUVECs. The levels of both p-eNOS (s1177) and eNOS were decreased in the D-gal group. NaHS treatment at 50 and 100 *μ*mol/L could reverse this trend (Figure 8(a)). We also checked eNOS mRNA expression. eNOS mRNA expression in HUVECs did not change after D-gal treatment, but it was increased with 50 *μ*mol/L NaHS supplementation (see Figure S1 in Supplementary Material available online at https://doi.org/10.1155/2017/5707830).

It is known that AKT phosphorylation is induced by NaHS in normal HUVECs. We asked whether AKT signalling is changed in cells undergoing accelerated ageing. We found that AKT phosphorylation, but not the total AKT, was decreased in the D-gal group. NaHS at 50 and 100 *μ*mol/L could induce the phosphorylation of AKT at Ser473, without altering the expression of total AKT (Figure 8(b)).

### 3.6. Effects of NaHS Treatment on Tumour Suppressor Expression in D-gal-Treated Mice and HUVECs

**Figure 9:**
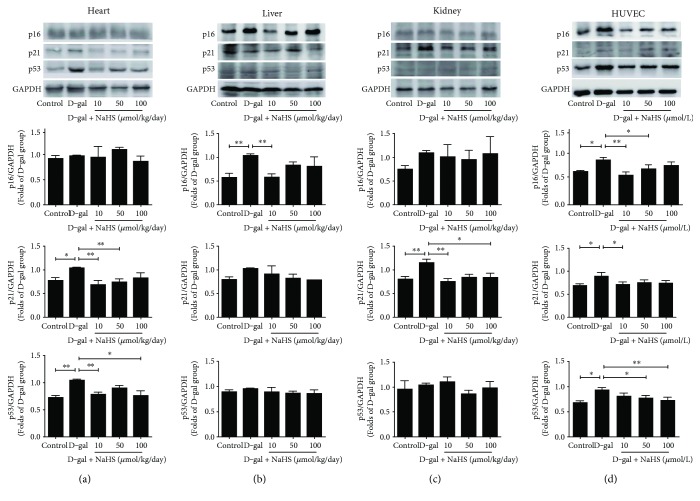
Influence of NaHS treatment on tumour suppressor expression in ageing mice and HUVECs. (a) p16, p21, and p53 expressions in the heart tissues. (b) p16, p21, and p53 expressions in the liver tissues. (c) p16, p21, and p53 expressions in the kidney tissues. (d) p16, p21, and p53 expression in HUVECs. *N* = 4. Values are the means ± SE. ^∗^*P* < 0.05 and ^∗∗^*P* < 0.01.

Oxidative stress upregulates the tumour suppressor p53 and its target genes, such as p21. The levels of p16 also increase in most ageing mammalian tissues. We checked p16, p21, and p53 expressions in D-gal-treated mice and HUVECs. Compared with the control, these three tumour suppressors were expressed at higher levels in the heart, liver, and kidney tissues after D-gal injection. More importantly, their expression was reduced after two months of NaHS treatment. In the heart tissue, sustained NaHS treatment at 10 and 50 *μ*mol/kg/day reduced p21 expression, while NaHS treatment at 10 and 100 *μ*mol/kg/day reduced p53 expression (Figure 9(a)). In the kidney, only NaHS treatment at 10 *μ*mol/kg/day reduced p16 expression (Figure 9(b)). In the liver, NaHS at 10 and 100 *μ*mol/kg/day was effective for p21 expression reduction (Figure 9(c)).

D-gal exposure for 48 h elevated all of the three tumour suppressor expressions in HUVECs. NaHS supplementation reduced their expression. The optimal dose for p16 reduction is 10 and 50 *μ*mol/L and for p21 is 10 *μ*mol/L, while for p53 is 50 and 100 *μ*mol/L (Figure 9(d)).

## 4. Discussion

In this study, we adopted an accelerated ageing model to investigate the effects of sustained NaHS treatment in the ageing process both in vivo and in vitro. Our work reveals two key findings: (1) lowered heart, liver, and kidney H_2_S levels, as well as lowered H_2_S-producing enzymes are associated with D-gal-induced accelerated ageing; (2) exogenous administration of the H_2_S donor NaHS mitigates age-related dysfunction, and the protective effects of NaHS may at least partially be due to improved endogenous H_2_S and NO production and the antioxidative ability of H_2_S.

Endogenous H_2_S has an essential role in internal environment homeostasis. Endogenous H_2_S production is insufficient in several diseases, such as hypertension and myocardial infarction [20, 21]. Enhanced endogenous H_2_S production in the heart tissue is believed to play a role in protecting the myocardium [20, 21]. We speculated that endogenous H_2_S levels might change during D-gal-induced ageing. The measurement of endogenous H_2_S levels confirmed decreased H_2_S levels in the heart, liver, and kidney tissues in D-gal mice. We also observed decreased CSE expression in the heart and kidney. Interestingly, sustained NaHS treatment for 2 months significantly elevated the expressions of CSE and CBS in the heart, liver, and kidney tissues. A major difference between the expression changes in CSE and CBS lies in the effective dosages of NaHS. NaHS supplementation at 50 and 100 *μ*mol/kg/day raised CBS expression in all three tissue types. With regard to CSE expression, NaHS treatment at both 50 and 100 *μ*mol/kg/day was effective in the heart tissues. Additionally, the NaHS dosage regimes of 50 and 100 *μ*mol/kg/day enhanced CSE expression in the liver and kidney, respectively. A certain degree of tissue specificity may result from the diverse distribution and abundant expression of H_2_S-producing enzymes in the given tissue types. For D-gal-challenged HUVECs, NaHS treatment elevated endogenous H_2_S production by increasing the expression of all three H_2_S-producing enzymes. Similar to previous reports, our findings indicate that age-related damage was inversely correlated with endogenous H_2_S production.

In the present study, all animals were freely fed with normal food. Additionally, D-gal-treated mice received hypodermic injections of 50 mg/kg D-gal daily for 2 months. Long-term administration of D-gal increases the concentration of intracellular D-gal. As a reducing sugar, D-gal reacts with the amino groups in various intracellular proteins and lipids [22]. The oxidative metabolism of D-gal generates advanced glycation end products (AGEs), and accumulated AGEs increase the production of ROS [22–24]. ROS generation is the major cause of intracellular damage induced by D-gal [23–25]. Natural ageing also exhibits high levels of oxidative stress [26, 27]. Exogenous H_2_S reverses D-gal-induced ageing through decreased ROS generation and lipid peroxidation, but the detailed mechanism is not clearly understood [28]. In D-gal-induced ageing, activities of the major endogenous antioxidant enzymes, such as SOD and GPx, are decreased. ROS scavenging or preventing its formation is beneficial in D-gal-induced ageing [29, 30]. Our previous studies in GK diabetic rats [25] and using the kidneys of naturally aged mice [18] indicate that sustained NaHS treatment significantly reduces ROS levels in vivo. In agreement with these findings, we found here that NaHS treatment could reduce ROS levels and increase GPx levels and SOD activity in accelerated ageing induced by D-gal both in vitro and in vivo. The protective effect of NaHS against D-gal-induced tissue injury is related to its antioxidant nature. Notably, there are similarities as well as differences between natural ageing and D-gal-induced ageing. For example, a comparison between natural and D-gal-induced ageing was conducted by studying age-related central auditory system changes [31]. Auditory dysfunction, oxidative stress, apoptosis rates, and pathological changes are similar in both senescence models. However, the auditory brainstem response threshold in the D-gal-induced ageing group does not differ from the vehicle control group [31]. Another comparative study suggests similar changes with differences in NMDA receptor expression [32]. Conservative interpretations of the data may be desirable when D-gal-induced ageing models are used to understand the natural ageing process.

Oxidative stress upregulates the tumour suppressor p53 and its target genes, such as p21 [33]. The mechanism of senescence usually involves the p16 or p53 tumour suppressor pathways [34]. In this study, we found that p16, p21, and p53 expressions are activated in the D-gal model mice and HUVECs, and NaHS treatment inhibited the expression of these tumour suppressors and helped reduce cellular senescence.

Interestingly, endogenous NO levels are also decreased in D-gal-accelerated ageing. Since NO is an important endothelial-derived vasodilator and cardioprotector, low NO levels may contribute to D-gal-induced tissue damages. The two gaseous signalling molecules NO and H_2_S are indispensable for diverse cellular and systemic functions [14, 35, 36]. Recent studies revealed that these two gaseous molecules may have redundant or overlapping physiological functions as well as pathophysiological roles by virtue of their actions on similar molecular targets [37]. However, when and how NO and H_2_S interact under physiological and disease conditions remains unclear. Our previous study first reported that the novel proangiogenic effect of H_2_S was dependent on Akt phosphorylation [38]. Altaany et al. showed that H_2_S induced p38 MAPK/Akt and eNOS phosphorylation, which was followed by increased NO production [39]. In this study, we found that H_2_S could induce Akt phosphorylation in D-gal-challenged HUVECs. Additionally, exogenous H_2_S increased both the expression of eNOS and its phosphorylation at S1177. As expected, the cellular NO content was elevated. When endogenous H_2_S production was inhibited pharmacologically, the cellular NO content decreased. We found that eNOS mRNA expression in HUVECs did not change after D-gal treatment, but it was increased with 50 *μ*mol/L NaHS supplementation. We speculate that eNOS protein stability may play a role in D-gal-caused NO level reduction, and NaHS supplementation may mitigate this reduction by influencing eNOS mRNA expression and/or eNOS protein stability. These data indicate crosstalk between the H_2_S and NO signalling pathways in D-gal-treated senescent cells. More studies are needed to reveal the mechanism of crosstalk between the NO and H_2_S signalling pathways. The elucidation of their relationship may improve our understanding of the pathogenic mechanisms underlying age-related diseases.

## 5. Conclusions

We demonstrated that endogenous levels of H_2_S were insufficient in D-gal-accelerated ageing, which was caused by the impaired expression of H_2_S-producing enzymes. Sustained exogenous H_2_S treatment could protect D-gal-accelerated ageing both in vitro and in vivo by reducing oxidative stress and increasing eNOS expression and NO contents as well as increasing endogenous H_2_S production.

## Supplementary Material

FIGURE S1. Influence of NaHS treatment on eNOS mRNA expression in ageing HUVECs. N=5. Values are the means ± SE. ∗P < 0.05.
