# Supplementary material for: H2S Donor NaHS Changes the Production of Endogenous H2S and NO in D-Galactose-Induced Accelerated Ageing
Source: Oxid Med Cell Longev. 2017 Apr 23;2017:5707830. doi: 10.1155/2017/5707830 (PMC5420433; doi:10.1155/2017/5707830)
Supplement: Supplementary file 1 — FIGURE S1. Influence of NaHS treatment on eNOS mRNA expression in ageing HUVECs. N=5. Values are the means ± SE. ∗P < 0.05. [file 5707830.f1.pdf]

FIGURE S1

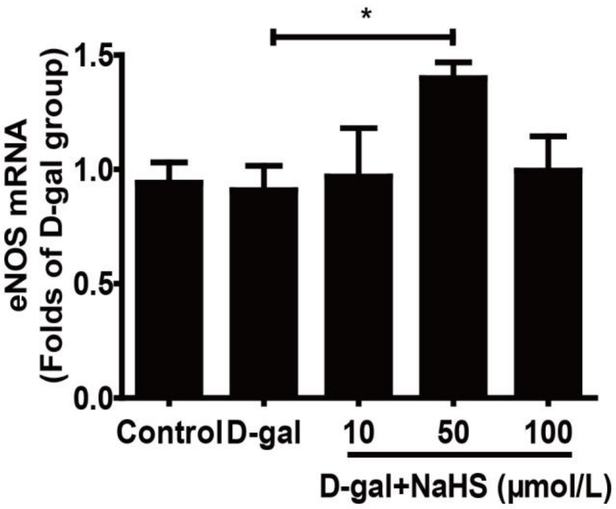

FIGURE S1. Influence of NaHS treatment on eNOS mRNA expression in ageing HUVECs. N=5. Values are the means ± SE. $*P < 0.05$.
